# Reluctance to Accept Palliative Care and Recommendations for Improvement

**DOI:** 10.1093/geroni/igab046.636

**Published:** 2021-12-17

**Authors:** Valeria Cardenas, Anna Rahman, Yujun Zhu, Susan Enguidanos

**Affiliations:** 1 University of Southern California, University of Southern California, California, United States; 2 University of Southern California, Los Angeles, California, United States; 3 University of Southern California, SOUTH PASADENA, California, United States

## Abstract

Although insurance companies are increasingly paying for home-based palliative care (HBPC), enrollment remains low. To identify patient and caregiver perceived barriers to HBPC and their recommendations for overcoming these barriers, we conducted semi-structured individual interviews. Our interview protocol elicited participants’ perspectives on HBPC services; positive and negative aspects of the palliative program explanation; and suggestions for improving HBPC messaging. Seventeen patients and eight caregivers who were eligible for a randomized controlled trial of HBPC were interviewed. Themes related to HBPC referral barriers included reluctance to have home visits, enrollment timing, lack of palliative care knowledge, and patients’ self-perceived health condition. Themes related to recommendations for overcoming these obstacles included ensuring that HBPC referrals come from healthcare providers or insurance companies and presenting HBPC more clearly. Findings reinforce the need for palliative care education among seriously ill patients and the importance of delivering palliative care information and referrals from trusted sources.

